# Endovascular Treatment of Cavernous Sinus Dural Arteriovenous Fistulas. Institutional Series, Systematic Review and Meta-Analysis

**DOI:** 10.1007/s00062-021-01107-0

**Published:** 2021-12-15

**Authors:** Andrea M. Alexandre, Carmelo Lucio Sturiale, Andrea Bartolo, Andrea Romi, Alba Scerrati, Maria Elena Flacco, Francesco D’Argento, Luca Scarcia, Giuseppe Garignano, Iacopo Valente, Emilio Lozupone, Alessandro Pedicelli

**Affiliations:** 1 UOC Radiologia e Neuroradiologia, Dipartimento di diagnostica per immagini, radioterapia oncologica ed ematologia, Fondazione Policlinico Universitario A. Gemelli IRCCS, Largo A. Gemelli 8, 00168 Roma, Italy; 2 Istituto di Radiologia, Università Cattolica del Sacro Cuore, Roma, Italy; 3 Dipartimento di Neurochirurgia, Fondazione Policlinico Universitario A. Gemelli IRCCS, Rome, Italy; 4 Dipartimento di Neurochirurgia, Ospedale Universitario S. Anna, Ferrara, Italy; 5 Dipartimento di Scienze Mediche, Università di Ferrara, Ferrara, Italy; 6 UOC Neuroradiologia, Ospedale V. Fazzi, Lecce, Italy; 7 Dipartimento di Morfologia, Chirurgia e Medicina Sperimentale, Università di Ferrara, Ferrara, Italy

**Keywords:** Cavernous sinus dural arteriovenous fistulas, Carotid-cavernous fistula, Carotico-cavernous d-AVF, DAVF, Endovascular treatment

## Abstract

**Purpose:**

Endovascular treatment represents the first-line therapy for cavernous sinus dural arteriovenous fistulas (CS-dAVF); however, different approaches and embolic agents as well as occlusion rates, complications and clinical outcomes are reported among the published series. In this study we performed a comprehensive meta-analysis to investigate clinical and radiological outcomes after endovascular treatment of CS-dAVFs.

**Methods:**

PubMed, Ovid Medline, Ovid EMBASE, Scopus, and Web of Science were screened for a comprehensive literature review from 1990 to 2020 regarding series of patients treated for CS-dAVF with endovascular approaches. We performed a proportion meta-analysis estimating the pooled rates of each outcome also including data of patients treated in our center.

**Results:**

A total of 22 studies reporting 1043 patients and 1066 procedures were included. Chemosis was reported in 559 out of 1043 patients (45.9%), proptosis in 498 (41.5%), and ophthalmoplegia in 344 (23.5%). A transvenous embolization was preferred in 753 cases (63.2%) and coils were used in 712 out of 1066 procedures (57.8%). Overall, 85% (95% confidence interval, CI 69.5–96.1%) of patients had a complete resolution of symptoms, while complications occurred in 7.75% (95% CI 3.82–12.7%) with minimal permanent deficits (0.15%). The mortality rate was 1 out of 1043 patients (< 0.001).

**Conclusion:**

A transvenous coiling is the most common endovascular approach for CS-dAVF, achieving a high percentage of radiological and clinical resolution and low complication rates. Transvenous approaches show less complications than transarterial ones, and coils appear safer than liquid embolic agents.

## Introduction

Dural arteriovenous fistula of the cavernous sinus (CS-dAVF) is an abnormal arteriovenous communication involving the dura mater of the CS wall. These fistulas represent about 16% of all cerebral dAVFs [1].

Endovascular treatment represents the first-line therapy in the literature [2, 3]; however, different approaches and embolic agents as well as occlusion rates, complications and clinical outcomes are reported among the published series. [4–6].

This study aims to systematically review all pertinent literature investigating clinical presentation, endovascular approach, embolizing agents, clinico-radiological outcomes and complications of endovascular treatments of CS-dAVFs.

## Material and Methods

### Study Design

This is a systematic review of the literature conducted according to the Preferred Reporting Items for Systematic Review and Meta-Analyses (PRISMA) statement. The review question was formulated according to the PICO criteria, as follows: (P, patients) in the management of CS-dAVF, (I, intervention) what is the endovascular treatment, (C, comparison) that reported the best results, (O, outcomes) in terms of clinical-radiologic outcomes.

This work is part of a non-profit study protocol approved from our hospital’s institutional ethics committee: protocol number 2477/21, ID 3585. Informed consent has been obtained from patients who participated in clinical investigations in our institutional series.

### Study Selection

PubMed, Ovid MEDLINE, Ovid EMBASE, Scopus, and the Web of Science were selected as online medical databases to conduct the present systematic review. The following search terms: “dural arteriovenous fistula”, “fistula”, “indirect”, “cavernous sinus” “carotid-cavernous” “carotid cavernous”, “carotico-cavernous”, “transarterial”, “transvenous”, “endovascular” were combined using the Boolean operators.

Studies reporting data on patients treated with endovascular techniques for CS-dAVFs were searched. We included all English articles reporting clinical and radiologic data for single patients published between 1990 and 2020. Clinical series reporting less than 10 patients, guidelines, reviews, commentaries, and letters to the editor were excluded.

The first-round search was conducted by 2 reviewers (A.R. and A.B.) who independently screened titles and abstracts for eligibility. The selected full texts and their reference lists (forward search) were screened and evaluated for inclusion in the second round.

In the third round, articles were screened for demographics, dAVF location, clinical onset, dAVF angioarchitectural grading, treatment modalities, occlusion rates, procedural failure, procedure-related complications, radiological clinical outcome at final follow-up.

The article was then excluded in the case of data unavailability, incomplete data, improper data reporting, or unavailability of single patient data (exclusion with a reason). In the fourth round, data were retrieved and added to a database for pooling and statistical analysis (inclusion). Any discordance was solved by consensus with the senior authors (A.M.A. and C.L.S.). In the case of missing data, authors of the respective studies were contacted by email.

Last search was launched in December 2020.

### Institutional Series

We included a retrospective series of patients treated in our center in the period 2009–2020 for CS-dAVFs. All neuroradiological data were retrieved from the institutional PACS, while clinical data were collected through the analysis of all digital records.

### Outcomes Measurement

For every collected patient we recorded: CS-dAVF location, angioarchitectural grading according to Barrow and Cognard classification [44, 45], treatment modalities (including approach and embolizing agent), postoperative radiological outcome (scored as: complete resolution, near complete resolution, partial resolution and failed) and clinical outcome at final follow-up (scored as: clinical resolution, partial benefit, persistent and worsened).

Immediate angiographic result at the end of the procedure was classified as: a) complete treatment—in the case of a complete occlusion of the fistula with no evidence of persistent arteriovenous shunt; b) near complete resolution—in the case of minimal residual arteriovenous shunt at final angiographic control (residual flow < 10%); c*)* partial resolution—in the case of persistence of a significant shunting flow; d) failure—when the residual flow appeared substantially unmodified compared with the preoperative status.

### Statistical Analysis

We performed meta-analyses of proportions to estimate the pooled rates of each outcome. Pooled estimates were not computed when the frequency of an outcome was reported in < 1% of the sample (only raw proportions and 95% confidence interval, CI, were reported in such cases). All the included studies were single-group analyses, and no outcome comparison between groups was available, thus no head-to-head meta-analyses were performed. We adopted a random-effect model to account for the interstudy heterogeneity by using Stata software, version 13.1 (Stata Corp., College Station, TX, USA).

## Results

### Study Selection and Characteristics

According to our search strategy, 1054 articles in English language were retrieved through the electronic literature search.

After abstract reading, 721 papers were primarily excluded, while 374 were assessed for eligibility and analyzed in detail as they met our inclusion criteria.

After full-text reading and forward search from the selected papers bibliography, 85 articles were excluded because 22 included less than 10 patients, 36 reported incomplete follow-up or clinical details, 18 did not include CS-dAVFs, 2 were case reports, 1 was an editorial, 2 were focused on technical aspects, and 4 focused on materials.

**Fig. 1:**
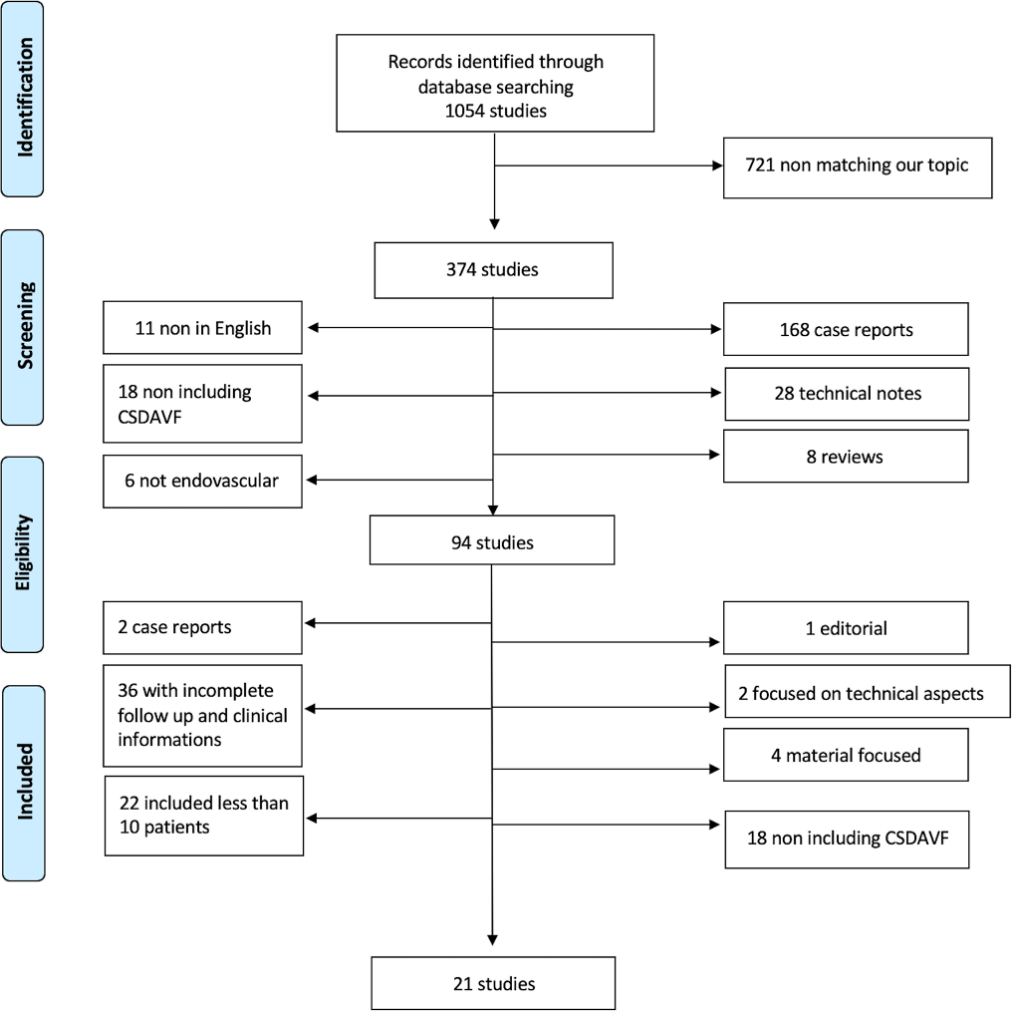
Search strategy flowchart

Subsequently 21 articles [5, 7–26] (19 retrospective, 1 prospective, and 1 mixed papers) reporting patients who underwent endovascular treatment for CS-dAVFs were finally included in this review (Fig. 1).

Finally, we included data from our unpublished series (Alexandre AM, 2021) in the statistical analysis. Details regarding included studies are reported in Table 1, whereas pooled proportions of the analyzed outcomes are reported in Tables 2 and 3. Finally, demographic, clinical and angioarchitectural data regarding our institutional series are reported in Table 4. In particular, we treated 17 patients (8 males, 9 female), with a mean age of 66 ± 11.7 years undergoing 21 procedures. Two patients, instead, showed a spontaneous symptoms resolution 1 week after steroids and anticoagulants therapy. All the other patients except one had a complete clinical resolution after treatment. As regards to treatment complications, 2 showed transient cranial nerve deficits spontaneously resolved after 3 months, and 1 had a minor ischemic embolic lesion.

**Table 1:** Characteristics of the included studies

First author	Publication year	Country	Study design	Total sample	% Males	Mean age, years (SD)	N. fistulas	N. procedures
Klisch	2003	Germany	Retrospective	11	18.2	62.5 (15.5)	14	11
Luo	2009	China	Retrospective	11	72.7	31.0 (NR)	11	11
Lv	2008	China	Retrospective	17	47.1	47.6 (NR)	20	22
Miller	1995	USA	Retrospective	10	40.0	51.9 (14.8)	11	10
Nishimuta	2017	Japan	Retrospective	50	16.0	66.2 (11.3)	59	50
Pashapour	2014	Iran	Prospective	46	63.0	36.8 (NR)	47	46
Satow	2013	Japan	Retrospective	20	25.0	67.5 (NR)	21	22
Yoshida	2009	Japan	Retrospective	44	29.5	66.0 (NR)	51	49
Zhang	2010	China	Retrospective	22	72.7	49.0 (NR)	22	27
Lee	2019	Korea	Retrospective	121	33.1	58.0 (11.9)	134	153
Rhim	2015	Korea	Retrospective	49	30.6	57.2 (NR)	49	49
Yu	2007	China	Retrospective	98	25.5	57.66 (17.2)	98	74
Alexander	2019	USA	Retro- and prospective	267	21.7	60.9 (NR)	267	267
Ertl	2020	Germany	Retrospective	33	30.3	65.5 (13.7)	33	33
Cha	2012	Korea	Retrospective	14	28.6	60.6 (9.4)	14	11
Cheng	2003	China	Retrospective	27	11.1	60.3 (12.9)	27	29
De castro-Afonso	2017	Brazil	Retrospective	62	38.7	62.7 (12.5)	62	63
Jiang	2008	China	Retrospective	12	25.0	54.5 (14.9)	12	12
Jia	2018	Korea	Retrospective	52	23.1	59.1 (10.7)	52	53
Holland	2018	Australia	Retrospective	23	21.7	64.0 (NR)	23	13
Griauzde	2016	USA	Retrospective	37	29.7	64.0 (NR)	37	32
Alexandre	2021	Italy	Retrospective	17	47.1	66.0 (11.7)	17	21

*NR* Not reported

**Table 2:** Pooled proportions of clinical symptoms, classification schemes and treatment strategies in patients with dural arteriovenous fistulas. Data from single studies have been combined using proportion meta-analysis (random-effect model)

Outcomes (22 studies included)	Patients (*n*/*N*)	Raw proportions (95% CI)	Pooled proportions (95% CI)	I^2^ (%)
*1. Barrow classification:*				
Type A	0/1043	–	–	–
Type B	110/1043	10.5 (8.74–12.6)	5.70 (1.51–11.7)	89
Type C	85/1043	8.15 (6.56–10.0)	7.79 (2.63–14.8)	90
Type D	434/1043	41.6 (38.6–44.7)	31.9 (12.5–55.0)	98
*2. Cognard classification:*				
Type I	45/1043	4.31 (3.16–5.73)	1.89 (0.0–5.72)	85
Type IIa	204/1043	19.6 (17.2–22.1)	17.7 (5.60–33.9)	97
Type IIb	43/1043	4.12 (2.99–5.51)	0.85 (0.0–4.02)	85
Type IIa + b	75/1043	7.19 (5.69–8.63)	4.85 (0.82–11.0)	90
Type III	10/1043	0.96 (0.46–1.77)	0.07 (0.0–0.85)	34
Type IV	14/1043	1.34 (0.74–2.24)	0.07 (0.0–1.10)	55
Type V	1/1043	0.09 (0.0–0.5)	–	–
*3. Fistula location:*				
Left	262/1043	25.1 (22.5–27.9)	21.3 (10.0–35.2)	95
Right	282/1043	27.0 (24.4–29.8)	23.3 (11.2–37.9)	96
Bilateral	91/1043	8.72 (7.08–10.6)	7.41 (2.45–14.2)	90
*4. Cortical reflux*	383/1043	36.7 (33.8–39.7)	26.5 (17.3–36.7)	91
*5. Clinical presentation:*				
Proptosis	498/1043	47.8 (44.7–50.8)	41.5 (20.9–63.5)	98
Ophtalmoplegia	344/1043	33.0 (30.1–35.9)	23.5 (7.80–43.8)	98
Pulsatile tinnitus	166/1043	15.9 (13.7–18.3)	10.6 (4.54–18.5)	90
Pain and/or headache	208/1043	19.9 (17.6–22.5)	11.1 (2.73–23.0)	96
Chemosis	559/1043	53.6 (50.5–56.7)	45.9 (22.0–70.7)	98
Visual acuity reduction	216/1043	20.7 (18.3–23.3)	12.4 (4.48–23.0)	94
Ocular symptoms	153/1043	14.7 (12.6–17.0)	4.96 (0.0–16.8)	97
Cranial nerve palsy	500/1043	47.9 (44.9–51.0)	31.2 (16.8–47.6)	96
Elevated IOP or glaucoma	146/1043	14.0 (11.9–16.3)	1.92 (0.0–9.03)	94
Focal neurological deficit	2/1043	0.19 (0.02–0.69)	–	–
*6. Embolization approach*^a^:				
Transarterial	220/1066	20.6 (18.2–23.2)	14.3 (7.75–22.3)	89
Transvenous	743/1066	69.7 (66.8–72.4)	63.2 (50.6–65.0)	93
Combined	63/1066	5.91 (4.57–7.50)	4.78 (1.80–8.72)	78
Direct puncture	1/1066	0.09 (0.0–0.5)	–	–
*7. Embolizing agent*^a^:				
Coils	712/1066	66.8 (63.9–69.6)	57.8 (45.4–69.7)	93
Glue	54/1066	5.07 (3.83–6.56)	2.78 (0.15–7.73)	87
EVOH	47/1066	4.41 (3.26–5.82)	2.97 (0.30–7.31)	86
Balloon	10/1066	0.94 (0.45–1.72)	–	–
PVA	28/1066	2.63 (1.75–3.77)	0.71 (0.0–2.31)	55
Liquid agents	55/1066	5.16 (3.91–6.63)	0.25 (0.0–2.47)	82
Coils + glue	33/1066	3.10 (2.14–4.32)	1.43 (0.0–4.32)	79
Coils + EVOH	32/1066	3.0 (2.06–4.21)	1.38 (0.0–4.54)	82
Others^b^	25/1066	2.35 (1.52–3.44)	0.19 (0.0–1.22)	43

*n* number of subjects with the outcome, *N* total number of subjects, *CI* confidence interval, *IOP* intraocular pressure, *EVOH* ethylene vinyl alcohol, *PVA* Polyvinyl alcohol

^a^The unit of analysis is the number of procedures (instead of number of patients)

^b^Including: stent; EVOH + balloon; stent + balloon; coils + balloon; glue + pva; coils + pva, balloon + stent + coils

**Table 3:** Pooled proportions of selected clinical and radiological outcomes in patients with dural arteriovenous fistulas. Data from single studies have been combined using proportion meta-analysis (random-effect model)

*Outcomes (22 studies included)*	Patients (*n*/*N*)	Raw proportions (95% CI)	Pooled proportions (95% CI)	I^2^ (%)
*1. Postsurgical radiological outcome:*				
Complete occlusion	599/1043	57.4 (54.4–60.5)	79.5 (63.0–92.3)	97
Near complete occlusion	176/1043	16.9 (14.6–19.3)	6.09 (1.16–13.5)	92
Partial occlusion	38/1043	3.64 (2.59–4.97)	2.02 (0.24–3.88)	74
Failed procedure	4/1043	0.38 (0.10–0.98)	–	–
*2. Clinical status at final follow-up:*				
Resolution	769/1043	73.7 (70.9–76.4)	85.0 (69.5–96.1)	97
Partial benefit	65/1043	6.23 (4.84–7.87)	2.45 (0.27–5.96)	81
Persistent	14/1043	1.34 (0.74–2.24)	0.19 (0.0–1.02)	23
Worsened	16/1043	1.53 (0.88–2.48)	0.22 (0.0–0.95)	9
*3. Complications onset*	95/1043	9.11 (7.43–11.0)	7.75 (3.82–12.7)	80

*n* number of subjects with the outcome, *N* total number of subjects, *CI* confidence interval

**Table 4:** Demographic, clinical and angioarchitectural characteristics of the included patients

Patient	Sex/Age (years)	Feeders	Drainage	Barrow type	Cognard type	Cortical reflux	Treatment access	N. of treatments	Embolic agent	Angiographic outcome	Clinical outcome
1	M/56	ICAbr, AMA, APA	IPS	D	IIa	No	TFV	1	Coils	Complete	Resolution
2	F/56	ICAbr, IMA	Sylvian vein/IPS	D	I	Yes	Autoresolution^a^	–	–	–	Resolution
3	F/78	ICAbr, AMA	CS-SOV/IPS	D	IIa	No	TFV/TA	2	Coils	Complete	Resolution
4	F/70	MMA, AMA	CS-SOV	C	IIa	No	TA/TFV	2 (1 Failed)	Onyx	Complete	Resolution
5	M/82	MMA, AMA, ICAbr	CS-SOV/IPS	D	IIa	No	TFV	1	Coils	Complete	Resolution
6	F/58	ICAbr, APA, MMA	CS-SOV	D	IIa	No	TA	1	Onyx	Near Complete	Resolution
7	M/67	AMA, ICAbr	CS-SOV	D	IIa	No	TA	1	PVA	Complete	Resolution
8	M/38	ICAbr, MMA, ECAbr	CS-SOV/IPS	D	IIa	No	TFV	1	Coils	Complete	Resolution
9	M/63	APA, ICAbr	CS-SOV	D	IIa	No	TFAV	1	Coils	Complete	Resolution
10	M/74	AMA, ICAbr	CS-SOV	D	IIa	No	TA	1	PVA	Complete	Resolution
11	M/67	APA, MMA, ICAbr	Sphenoparietal sinus	D	III	Yes	TA	2	Onyx	Complete	Resolution
12	F/76	ICAbr, APA, MMA, AMA	CS-Contralateral SOV	D	IIa	No	TFV/TA	2 (1 Failed)	Coils	Complete	Resolution
13	F/80	ICAbr	SOV	B	IIa	No	Autoresolution^a^	–	–	–	Resolution
14	F/79	AMA, APA, ECAbr, ICAbr	SOV/IPS	D	IIa + b	No	TFV	1	Coils	Complete	Partial benefit
15	F/53	ICAbr, IMA	SOV/IPS	D	IIa	No	TFV/TA	2	Coils/PVA	Complete	Resolution
16	F/65	IMA	SOV/IPS	C	IIa	No	TFV	1	Coils	Complete	Resolution
17	M/61	IMA, ICAbr	SOV/IPS	D	IIa	No	TFV/TA	2	Coils/PVA	Complete	Resolution

*ECAbr* multiple branches from external carotid artery, *ICAbr* dural branches from internal carotid artery, *IPS* inferior petrosal sinus, *CS-SOV* cavernous sinus-superior ophthalmic vein, *CS* cavernous sinus, *MMA* middle meningeal artery, *AMA* accessory meningeal artery, *APA* ascending pharyngeal artery, *IMA* internal maxillary artery, *TFV* transfemoral vein, *TA* transarterial, *TFAV* trans-facial vein, *PVA* polyvinyl alcohol embolization particles

^a^Spontaneous resolution of symptoms after steroids and anticoagulants.

### Demographic Characteristics

Overall, we collected 1043 patients suffering from 1081 CS-dAVFs, who underwent a total of 1066 endovascular procedures. Among them, 311 were males (29.8%) and 732 females (70.2%). Mean age was 57.6 years (standard deviation was reported in a minority of series).

### dAVFs Characteristics

According to Barrow classification, 110 patients had a type B fistula (5.7%), 85 a type C (7.8%) and 434 a type D (31.9%), while in 414 patients the fistula type was not reported. Considering Cognard classification, 45 patients (1.9%) had a type I fistula, 204 a type IIa (17.7%), 43 a type IIb (0.8%), 75 a type IIa + b (4.8%), 10 a type III (0.07%), 14 a type IV (0.07%), 1 a type V, while in 651 cases it was not reported. The fistula was located on the right side in 282 patients (21.3%), on the left in 262 (23.3%), bilaterally in 91 (7.4%), while the side was not reported in 408 (Table 2).

### Clinical Presentation

Chemosis was the most frequent clinical presentation, reported in 559 out of 1043 patients (45.9%), followed by proptosis in 498 (41.5%), ophthalmoplegia in 344 (23.5%), pulsatile tinnitus in 166 (10.6%), pain and/or headache in 208 (11.1%), visual acuity reduction in 216 (12.4%), unspecified ocular symptoms in 153 (4.96%), cranial nerve palsy in 500 (31.2%), an elevated intraocular pressure or glaucoma in 146 (1.92%), a focal neurological deficit was the less common presentation, reported in 2 patients (< 0.01%—Table 2).

### Type of Endovascular Procedure

The type of embolization approach was reported in 1037 out of 1066 procedures (97.2%) summarized in Table 2.

A transvenous embolization was used in 753 procedures (63.2%), a transarterial embolization in 220 (14.3%), a combined approach in 63 (4.78%), while a direct puncture in 1 case (< 0.01%). In 36 cases out of 1043, a conservative treatment allowed the symptoms resolution in 16 patients (1.5%).

Considering embolizing agents, coils were used in 712 out of 1066 procedures (57.8%), glue in 54 (2.78%), ethylene vinyl alcohol (EVOH) in 47 (2.97%), balloon in 10 (< 0.01%), polyvinyl alcohol particles (PVA) in 28 (0.7%), liquid agents in 55 (0.25%), coils plus glue in 33 (1.43%), coils plus EVOH in 32 (1.38%), while other alternatives including EVOH plus balloon, stent plus balloon, coils plus balloon, glue plus PVA, coils plus PVA, balloon plus stent and coils were overall used in 25 cases (0.19%).

### Radiological Outcome

Immediate radiological outcome was reported in 817 out of 1043 (78.3%) patients. A complete resolution of the fistula was obtained in 79.5% (95% CI, 63.0–92.3%), a near-complete 6.09% (95% CI, 1.6–13.5%), a partial obliteration in 2.02% (95% CI, 0.24–3.88%), while a failure was experienced in 4 patients (< 0.01%, Table 3).

### Clinical Outcome and Complications

Details are reported in Table 3: clinical outcome at final follow-up was reported in 864 out of 1043 (82.8%) patients. Of these, 85% (95% CI, 69.5–96.1) had a complete resolution of symptoms, 2.45% (95% CI, 0.27–5.96) had a partial benefit, 0.19% (95% CI 0.0–1.02) a persistent symptomatology, and 0.22% (95% CI 0.0–0.95) a worsening of the clinical status.

Mean time of follow-up was reported in 21 out of 22 papers, and it was about 22.2 months.

Complications occurred in 7.75% (95% CI 3.82–12.7) of patients. Out of 95 complications 66 (6.3%) led to a transient deficit, while 16 (0.15%) led to a permanent one. Among the severe complications, there were 3 cases of intracerebral or subarachnoid hemorrhage, 7 of new or persistent cranial nerve palsy, 6 of cerebral ischemia. Overall, mortality rate was 1 out of 1043 patients (< 0.001).

## Discussion

This systematic review and meta-analysis including 22 studies on endovascular treatment of CS-dAVF showed that the most frequent clinical onset is characterized by ocular symptoms, essentially chemosis (45.9%), proptosis (41.5%) and oculomotor nerve palsy (31.2%). A transvenous approach was preferred by the authors in almost 2/3 of cases (63.2%) and coils were used in more than half of patients. In general, the endovascular approach demonstrated a high percentage of radiological (79.5%) and clinical (85%) resolution of the fistula, and a low complication rate (< 8%), with an almost negligible incidence of permanent deficit.

### Clinical Presentation

CS-dAVFs often present with various combinations of orbital signs and symptoms, which occur when the fistulous venous drainage involves the ophthalmic veins; however, venous congestion can seldom manifest with vague cognitive symptoms, such as concentration disorders [18].

Sometimes, CS-dAVFs may be associated with severe morbidity like threatening blindness, stroke and cerebral hemorrhage, especially if they are characterized by retrograde venous drainage within the cortical venous system [4, 27].

Our meta-analysis showed that the most frequently presentation symptoms were chemosis (45.9%), proptosis (41.5%) and ophthalmoplegia (23.5%). Oculomotor palsy was reported in almost 1/3 of patients (31.2%): among them, a VI cranial nerve palsy was objectivated in about 4/5, probably due to its inner course within the cavernous sinus, while a III and IV cranial nerves palsy overall in 1/5.

On the other hand, local pain, headache, pulsatile tinnitus and visual acuity reduction were less frequently reported; however, whether the first three may be relatively subjective symptoms, visual impairment and increase of the intraocular pressure reflect a condition of severe venous congestion observed in advanced cases [28, 29].

Anyway, clinical manifestations remain unpredictable in several cases without a precise explanation. Nowadays, the most accredited interpretation has been given by Stiebel-Kalish et al., who suggested that the venous drainage pattern may be responsible for the different clinical onset in this type of fistulas [30].

### Endovascular Treatment

The goal of CS-dAVFs treatment is to reduce cavernous sinus pressure by interrupting the fistulous communications and the retrograde flow towards the cortical venous system [6, 29, 31]. Before the advent of neurointerventional techniques, therapeutic options were limited [32], and in some cases a conservative management were preferred. Moreover, we found that the resolution of the fistula with a conservative treatment (e.g. manual carotid artery compression, steroid therapy) occurs as rarely as about 1.5% out of cases [33], but we cannot exclude that these were fistulas with a very low-flow with a spontaneous self-limiting behavior.

**Fig. 2:**
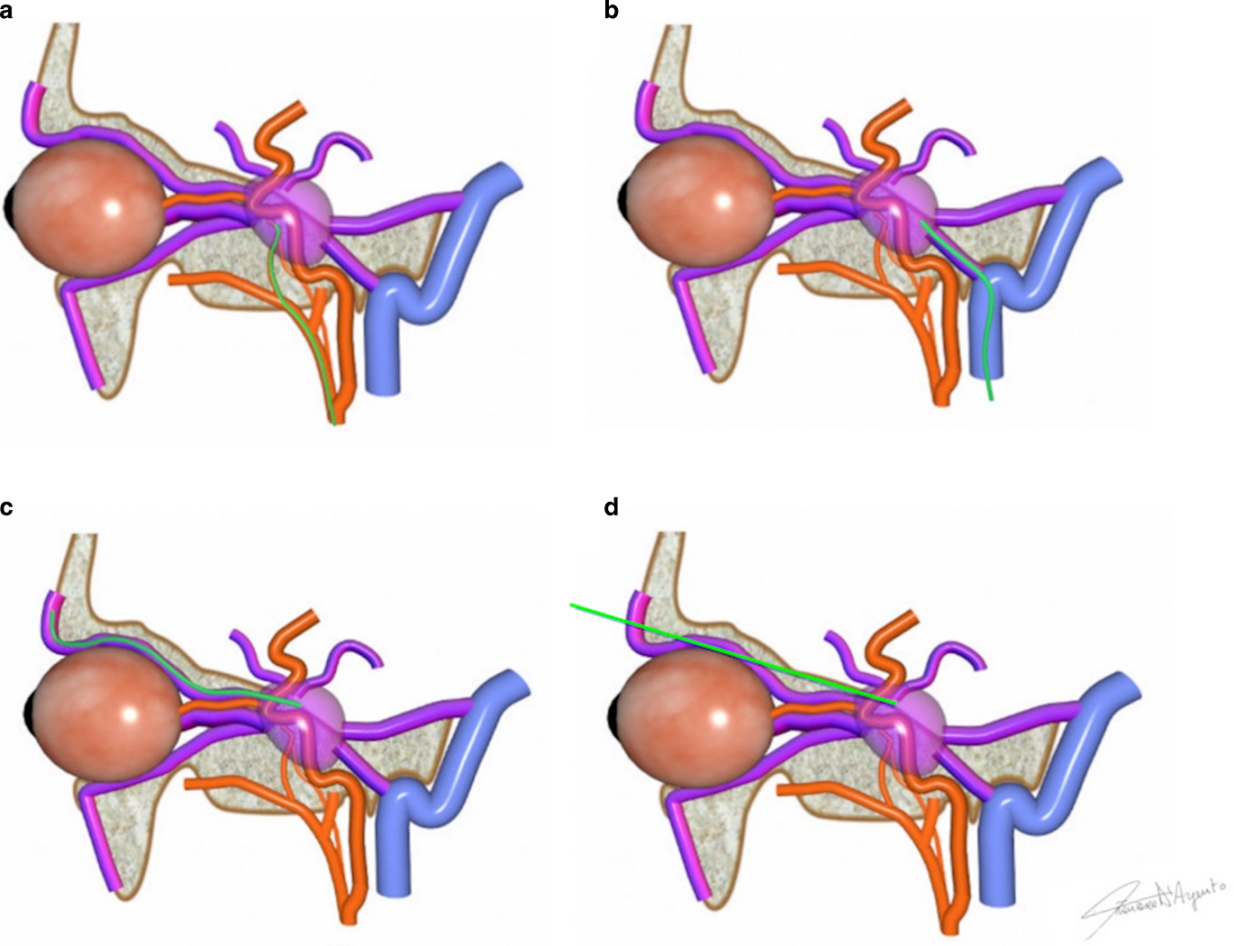
Different approaches for endovascular treatment of CS-dAVF. **a** Arterial approach, **b** transvenous through inferior petrosal sinus approach, **c** transvenous through superior ophthalmic vein approach, **d** direct puncture of the cavernous sinus through the superior ophthalmic fissure

The preferred approach in literature is the transvenous embolization of the CS affected by the fistula [6], commonly acceding through the inferior petrosal sinus via the internal jugular vein ([4, 6]; Fig. 2).

Although some authors reported a certain success with transarterial approaches with liquid embolic agents, most of them preferred a transvenous approach releasing coils into the cavernous sinus. This approach, in fact, obviates the need for catheterization and embolization of the multiple tiny arterial feeders usually supplying the CS-dAVF [6, 31, 34, 35], reducing the high risk of ischemic complications due to dangerous extracranial-intracranial anastomoses, or cranial nerve palsy due to vasa nervorum occlusion.

In agreement, we found that almost 2/3 of the procedures were performed with transvenous access (63.2%) compared with those performed with transarterial or combined approaches. Consequently, coils were the most frequently used embolizing agent (57.8%), whereas other embolizing agents, such as glue, EVOH, PVA or various combinations of these, were used only in cases of transarterial or combined accesses.

Direct punctures of various head veins, such as the superior orbital vein, facial vein, sylvian vein, or directly the cavernous sinus through the superior orbital fissure have been proposed [36–40], but all these approaches are challenging and generally reserved for particular cases in which no other pathway to the fistula is accessible.

### Alternative Treatment Options

Three main options are considered alternative to the interventional treatment for CS-dAVF. The first one is the conservative approach, which can be considered when symptoms are mild, no cortical venous drainage is present, and the angiographic assessment reveals a low-flow shunt. This includes medicinal management (prostaglandin analogues to control intraocular pressure, analgesics, steroids and anticoagulants to avoid occlusion of the superior ophthalmic vein), manual compression therapy and controlled hypotension. Occasionally a spontaneous occlusion may be observed without any medication [41].

The second is radiotherapy, which is suggested by some authors for selected cases where the endovascular treatment appeared unsuccessful, ineffective, contraindicated or considered too risky. Both irradiation with linear accelerator or gamma knife are reported, but this technique should be preferred in case of low-flow shunts and combined with transarterial embolization or manual compression as its efficacy in high-flow lesions remains questionable.

Finally, surgical occlusion can be proposed only to facilitate endovascular approaches, in selected cases [41].

### Complications

According to the literature, complication rates appear variable, ranging from 2% to 20% [4, 32, 42], and some of them may result in significant morbidity.

In this meta-analysis, we found a pooled treatment-related complication rate of 7.75%, which were for the majority transient; only a small number (16/1043) showed permanent deficits after treatment.

This appeared in accordance with previous studies reporting III, IV, V1, V2 or VI cranial nerves deficit as the most frequent transient complications [42]. Probably the occurrence of a new cranial nerve deficit or a worsening of a previous one after treatment often reflects an acute thrombosis of the cavernous sinus. Nevertheless, other authors suggested that CS coils overpacking represents the predominant cause of posttreatment cranial nerve palsy [43]. In any case, coil embolization has been associated with fewer complications than liquid embolic agents [5, 18].

Compared with transvenous embolization, transarterial approach has been associated with higher complication rate [18].

### Clinical and Radiological Follow-up

Reported rates of complete CS-dAVFs occlusion after endovascular treatment range from 70% to 90%. In the included studies, clinical and radiological follow-up were reported in different ways, sometimes in terms of time intervals, sometimes in terms of instrumental tests. The mean follow-up time ranged from 3 to 67 months (mean 22 months).

A follow-up angiographic evaluation was reported in about 4/5 out of cases, documenting a pooled proportion of complete/near complete occlusion in more than 75% of patients.

In agreement, about 85% of patients showed a complete resolution of symptoms after treatment, with a negligible pooled percentage of patients showing symptoms persistence or worsening.

### Limitations

Our study presents several limitations: clinical and radiological follow-up times were variable (3–67 months) thus, follow-up data may be biased by a high heterogeneity. All the included papers were observational, non-randomized, and non-comparative studies and data were often lacking in several details.

Finally, our results could be influenced by publication bias. In fact, we could miss some studies with worse outcomes that were performed and not published, distorting the evidence base; however, this study provides useful data to consider when dealing with endovascular treatment of CS-dAVFs.

## Conclusion

A transvenous coiling is the most used endovascular treatment, achieving a high percentage of radiological and clinical resolution with a very low complication rate. In general, transvenous treatments showed less complications than transarterial approaches, and coil occlusion appeared safer than using liquid embolic agents.
